# BubR1 Acts as a Promoter in Cellular Motility of Human Oral Squamous Cancer Cells through Regulating MMP-2 and MMP-9

**DOI:** 10.3390/ijms160715104

**Published:** 2015-07-03

**Authors:** Chou-Kit Chou, Chang-Yi Wu, Jeff Yi-Fu Chen, Ming-Chong Ng, Hui-Min David Wang, Jen-Hao Chen, Shyng-Shiou F. Yuan, Eing-Mei Tsai, Jan-Gowth Chang, Chien-Chih Chiu

**Affiliations:** 1 Graduate Institute of Natural Products, Kaohsiung Medical University, Kaohsiung 807, Taiwan; E-Mail: fatchou1988@hotmail.com; 2 Department of Biotechnology, Kaohsiung Medical University, Kaohsiung 807, Taiwan; E-Mails: yifuc@kmu.edu.tw (J.Y.-F.C.); mincong2000@gmail.com (M.-C.N.); 3 Department of Biological Sciences, National Sun Yat-sen University, Kaohsiung 804, Taiwan; E-Mail: cywu@mail.nsysu.edu.tw; 4 Department of Fragrance and Cosmetics Science, Kaohsiung Medical University, Kaohsiung 807, Taiwan; E-Mail: davidw@kmu.edu.tw; 5 Center for Stem Cell Research, Kaohsiung Medical University, Kaohsiung 807, Taiwan; 6 School of Dentistry, College of Dental Medicine, Kaohsiung Medical University, Kaohsiung 807, Taiwan; E-Mail: jehach@kmu.edu.tw; 7 Translational Research Center, Kaohsiung Medical University Hospital, Kaohsiung Medical University, Kaohsiung 807, Taiwan; E-Mail: yuanssf@ms33.hinet.net; 8 Department of Obstetrics and Gynecology, Kaohsiung Medical University Hospital, Kaohsiung Medical University, Kaohsiung 807, Taiwan; 9 Research Center for Environment Medicine, Kaohsiung Medical University, Kaohsiung 807, Taiwan; E-Mail: tsaieing@kmu.edu.tw; 10 Epigenome Research Center, China Medical University Hospital, Taichung 404, Taiwan; 11 Graduate Institute of Integrated Medicine, College of Chinese Medicine, China Medical University, Taichung 404, Taiwan; 12 Department of Laboratory Medicine, China Medical University Hospital, Taichung 404, Taiwan

**Keywords:** BubR1, spindle assembly checkpoint, oral squamous cancer cell, migration, metastasis

## Abstract

BubR1 is a critical component of spindle assembly checkpoint, ensuring proper chromatin segregation during mitosis. Recent studies showed that BubR1 was overexpressed in many cancer cells, including oral squamous cell carcinomas (OSCC). However, the effect of BubR1 on metastasis of OSCC remains unclear. This study aimed to unravel the role of BubR1 in the progression of OSCC and confirm the expression of BubR1 in a panel of malignant OSCC cell lines with different invasive abilities. The results of quantitative real-time PCR showed that the mRNA level of BubR1 was markedly increased in four OSCC cell lines, Ca9-22, HSC3, SCC9 and Cal-27 cells, compared to two normal cells, normal human oral keratinocytes (HOK) and human gingival fibroblasts (HGF). Moreover, the expression of BubR1 in these four OSCC cell lines was positively correlated with their motility. Immunofluorescence revealed that BubR1 was mostly localized in the cytosol of human gingival carcinoma Ca9-22 cells. BubR1 knockdown significantly decreased cellular invasion but slightly affect cellular proliferation on both Ca9-22 and Cal-27 cells. Consistently, the activities of metastasis-associated metalloproteinases MMP-2 and MMP-9 were attenuated in BubR1 knockdown Ca9-22 cells, suggesting the role of BubR1 in promotion of OSCC migration. Our present study defines an alternative pathway in promoting metastasis of OSCC cells, and the expression of BubR1 could be a prognostic index in OSCC patients.

## 1. Introduction

Death due to head and neck cancer has been on the rise in recent decades, accounting for around 580,000 cancer-related deaths worldwide in 2013 [1]. Oral cancer is one of most common types of head and neck cancer with poor prognosis and low survival rate. It could be found in any part of the oral cavity, but primarily appears on the tongue and floor of the mouth [2]. Over 90% of oral cancers are subjected to oral squamous cell carcinoma (OSCC), which exhibit as a neoplastic lesion with a tendency to extensively invade into the surrounding tissue [3]. Moreover, the occurrence of tumor metastasis in cervical lymph nodes is detected in about 40% of OSCC patients [4].

Metastasis is a multistep process that begins with the local invasion of cancer cells, which can overcome the extracellular matrix (ECM) and invade adjacent normal tissue. Meanwhile, invading cancer cells release matrix metalloproteinases (MMPs) to remodel the space of extracellular matrix, thus promoting cancer invasion [5]. In addition, MMP-2 and MMP-9, the two zinc-dependent endopeptidases with collagenase and gelatinase activity, have been reported to have high expression at the invasive front of OSCC tumor [6]. Although it is often asymptomatic at early stages, the appearance of neck lymph node metastasis seems to be a prognostic determinant for patients with OSCC [7]. Therefore, the examination of the neck of patients after receiving primary tumor resection is important. There are many available effective screening methods for neck metastasis, such as computed tomography (CT), magnetic resonance imaging (MRI) and positron emission tomography (PET). However, they are not enough to accurately diagnose the metastasis in cervical lymph nodes [8]. To improve the efficacy of the neck evaluation in patients with OSCC, it is urgent to identify a novel biomarker for predicting nodal metastasis from primary sites of oral tumors.

BubR1 (BUB (budding uninhibited by benomyl)-related 1), also called BUB1B or Mad3, is a core member of the spindle assembly checkpoint (SAC) complex, which acts as a guardian to monitor the attachment of kinetochores and microtubules during mitosis. BubR1 has three major functional domains: (1) an N-terminal region that contains two KEN box essential for competing with anaphase-promoting complex or cyclosome (APC/C) for binding to cell division cycle 20 (CDC20); (2) an intermediate region identified to be BUB3 binding site, which is crucial for BubR1 kinetochore localization; and (3) a C-terminal region that contains a catalytic serine/threonine kinase domain [9]. Moreover, decrease of more than 50% of BubR1 expression has been associated with premature chromatid separation (PSC), a rare autosomal disorder with a high risk of cancer incidence during infancy [10]. Down-regulation of BubR1 by oncogenic protein breast cancer-specific gene 1 (BCSG1)-mediated inhibition has also been observed in advanced stage breast cancer and is thought to promote chromosomal instability (CIN) [11].

Paradoxically, other reports have suggested that BubR1 overexpression leads to high incidence of aneuploidy coupled with malignant progression [12]. Based on the above studies, it appears that BubR1 at basal level functions to prevent missegregation of sister chromatids during mitosis, but either gain or loss of BubR1 expression would promote CIN-driven tumorigenesis and cancer progression. Therefore, it is essential to confirm whether BubR1 might be a prognostic marker in certain types of cancer. Recently, immunohistochemical studies of human oral mucosal SCC provide convincing evidence that overexpression of BubR1 is associated with OSCC tumorigenesis [13]. A similar finding also suggests that BubR1 might be a prognostic marker in OSCC patients [14]. In this study, we investigated the role of BubR1 in OSCC cell lines. Additionally, the effects of BubR1 on metastasis-related hallmarks, including the activity of MMP-2 and MMP-9, were examined and discussed.

## 2. Results and Discussion

### 2.1. Expression of BubR1 in Oral Squamous Cell Carcinoma (OSCC) Cells

**Figure 1:**
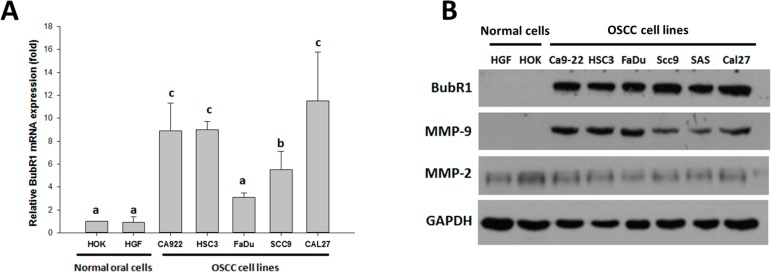
The levels of BubR1 in OSCC cell lines and normal cells. (**A**) Total RNA was collected after the cells incubation for 24 h. Quantificative analysis of BubR1 mRNA in various OSCC cell lines, and in both normal human oral keratinocytes (HOK) and human gingival fibroblasts (HGF), was examined by a quantitative reverse transcription PCR. Error bars show standard deviation of three independent experiments in duplicate. a *vs*. a, >0.05; a *vs.* b, <0.05; a *vs.* c, <0.001; (**B**) The protein expression of BubR1, MMP-9, and MMP-2 in a panel of OSCC cell lines compared to normal human oral keratinocytes (HOK) and human gingival fibroblasts (HGF).

It has been well documented that the expression levels of BubR1 is essential for monitoring the chromosome segregation. Abnormally expressed BubR1 would accelerate the possibility of tumorigenesis [12,15]. The levels that are fundamentally sufficient for BubR1 to operate in mitotic checkpoint are likely to arise with frequency during OSCC progression [14,16]. To confirm the relevance about promotion to a malignant state of OSCC could be accelerated by increasing of BubR1 levels, real-time PCR analyses were performed to investigate the mRNA levels of BubR1 in five OSCC cell lines, all of which have differential carcinomatous state with confirmed invasive ability, compared to normal primary human oral keratinocytes (HOK) and human normal gingival fibroblasts (HGF). The BubR1 mRNA levels in the OSCC cell lines were significantly higher than both normal cells except in FaDu cell line (Figure 1A). Meanwhile, this BubR1 levels in those of tested OSCC cell lines were quite consistent with their respective ability of cellular invasion that had been examined previously by using transwell assays [17]. We also assessed the protein expression of BubR1 in those above oral cell lines, and there is an association between decreased expression of BubR1 and matrix metalloproteinases-9 (MMP-9) in both normal oral cells and cancer cell lines (Figure 1B). However, the BubR1 levels positively correlated with the strength of invasiveness in OSCC seems to reflect the actions of BubR1 in promoting the OSCC metastasis, implying the coordination of MMP-9 and BubR1 in OSCC.

### 2.2. BubR1 Localizes Both in the Nuclear and Cytosol of OSCC Cells

**Figure 2:**
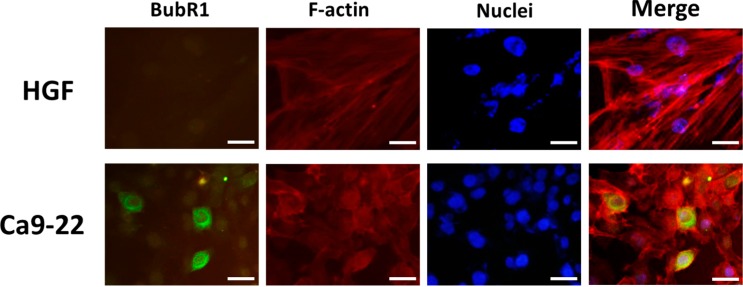
Intracellular distribution of BubR1 in normal oral fibroblast and OSCC cells. Ca9-22 and human normal gingival fibroblasts HGF were assessed for detecting the cellular localization of BubR1. Cells were treated with BubR1 antibody (green), Alexa Fluor^®^ 594 phalloidin for staining F-actin (red) and DAPI (blue). Magnification 200×. Scale bars represent 50 μm.

To detect whether the subcellular location of BubR1 would differ in carcinomatous OSCC cells and normal oral cells, the immunofluorescence assay was performed. The results showed that the endogenous BubR1 proteins are significantly overexpressed in Ca9-22 cells, and their localization seem to be localized around the nuclei of Ca9-22 cells rather than appearing within the expected nuclear space where BubR1 used to contribute in mitotic checkpoint. Even though not all the Ca9-22 cells exhibit in this dramatic form, however, the average of fluorescence intensity of BubR1 in Ca9-22 cells was higher than normal HGF cells (Figure 2). Previous study has demonstrated that the BubR1 functions in the mitotic phase to perform its surveillance that prevents the errors of chromosome segregation. Paradoxically, this tumor suppressor-liked BubR1 has been reported that its level is associated with cancer prognosis [18,19]. In comparison of the guardian role of BubR1 in mitotic checkpoint in normal cells, our observation suggested that the cytosolic accumulation of BubR1 might be more likely associated with the progression of OSCC tumorigenesis.

### 2.3. The Effect of BubR1 Knock-Down on Cell Morphology and Proliferation of OSCC Cells

**Figure 3:**
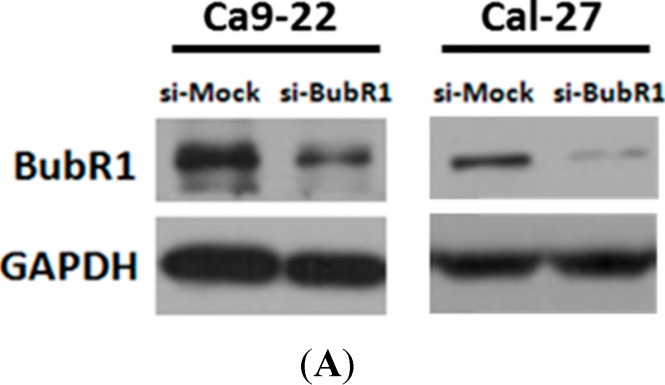

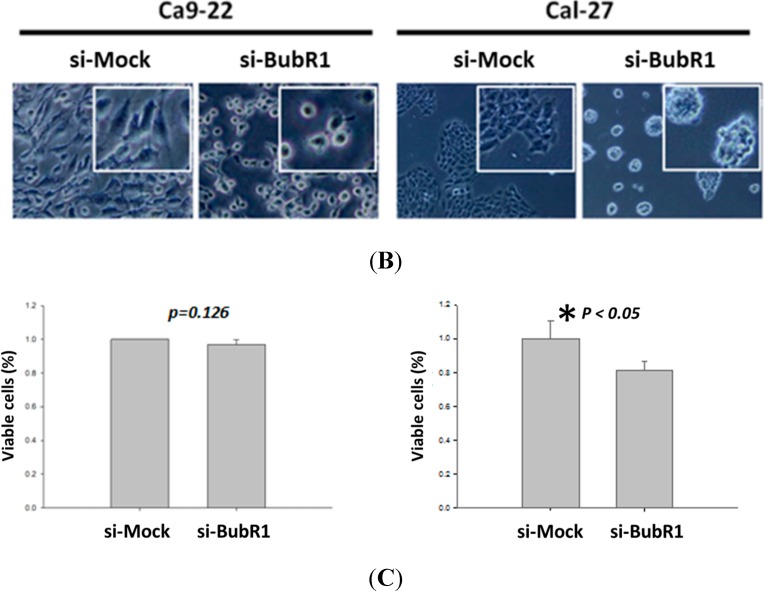
The effects of BubR1 knockdown on morphology and cell growth of OSCC cells. (**A**) The results of Western blot analysis confirmed the knockdown efficiency of BubR1 siRNA in two OSCC cell lines Ca9-22 and Cal-27; (**B**) The morphological changes of OSCC cells with BubR1 knockdown and (**C**) the cellular proliferation of Ca9-22 cells transfected with si-Mock or si-BubR1, respectively, were assessed using trypan blue exclusion assay. Each representative blot was performed in at least triplication. *p* = 0.126 for Mock *vs*. siRNA-BubR1, showing no statically significance between the two groups.

To analyze the function of BubR1 in OSCC cells, we transiently transfected siRNA oligonucleotide duplexes, which targeted the mRNA of BubR1 into Ca9-22 cell lines. Our results showed that the endogenous BubR1 expression was efficiently suppressed by siRNA as indicated by si-BubR1, whereas si-Mock served as a negative control (Figure 3A). We found that loss of BubR1 causes significantly morphological changes, including cells clumped tightly together and the appearance of cells are cobblestone-like, a hallmark of epithelial-type cells (Figure 3B). To test whether cell proliferation was affected by the levels of BubR1, viable cell counting was performed by using trypan blue staining after Ca9-22 cells were transfected siRNA for 48 h. Nevertheless, there was no difference in cell proliferation between the cells transiently transfected with si-Mock and si-BubR1 in Ca9-22 cells (Figure 3C). To further confirm the role of BubR1 on OSCC cells, siRNA-mediated knockdown approach performing in another OSCC cell line Cal-27 was used to conduct whether BubR1 takes effects on OSCC cells. The results showed that BubR1 knockdown attenuates cell proliferation of Cal-27 cells with about 20% reduction (* *p* *<* 0.05) (Figure 3C). Therefore, our present work showed that BubR1 knockdown might affect both the proliferation rate and cellular migration of OSCC cells. However, our present results suggest that effect of BubR1 knockdown on cellular migration rather than the cellular proliferation rate in OSCC cells.

### 2.4. BubR1 Knockdown Reduces the Invasive Ability of OSCC Cells

**Figure 4:**
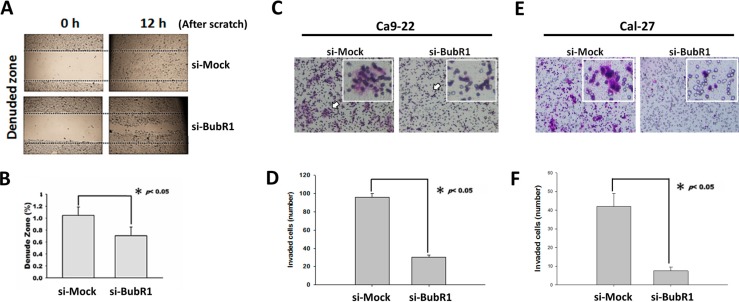
Knockdown of BubR1 causes a decreased migration ability of OSCC cells. (**A**) The effects of siRNA against BubR1 on cell migration. After seeding for 24 h to allow the transfected cells to form a full monolayer, the cells were scratched to create a wound area; (**B**) The migrating cells within the scratched were analyzed using a software “TScratch” [20]. The quantitative results of migrated wound site. Data are shown as means ± SD (*n* = 3). * *p* < 0.05 for Mock *vs*. siRNA-BubR1; (**C**,**E**) The effects of siRNA against BubR1 on both Ca9-22 and Cal-27 cell invasion. A total of 1 × 10^5^ transfected cells were seeded onto a permeable membrane in a Boyden chamber to allow the cells to invade the opposite layer of the membrane. Arrows point represents a cluster of invaded cells; and (**D**,**F**) The quantitative results of invaded cells. Data are shown as means ± SD (*n* = 3). * *p* < 0.05 for Mock *vs.* siRNA-BubR1.

To determine whether the levels of BubR1 could be a representative index of malignancy of OSCC cells, we used siRNA to transiently knockdown the mRNA level of BubR1 in Ca9-22 cells. In addition, metastasis is the worst threat to life of patients who are burdened with a malignant tumor. Therefore, we tested whether knockdown of BubR1 would affect the metastatic ability of Ca9-22 cells, particularly by wound healing assay, transwell invasion assays, and measuring the activity of MMP-2 and -9. These above experiments denoted the distinct steps to the overall process of cancer metastasis. We observed that knockdown of BubR1 only slightly retarded the Ca9-22 cells migration for reconstructing the scratched wound (Figure 4 A,B). Moreover, the transwell invasion assay showed that BubR1 knockdown significantly reduced the invaded cells more than 60% in Ca9-22 cells (Figure 4C,D). Likewise, the invasiveness of Cal-27 cells was almost abrogated after BubR1 silencing (Figure 4E,F), suggesting BubR1 might be essential for the progression of OSCC malignant tumor.

### 2.5. Knockdown of BubR1 Attenuates the Activity of MMP-2 and MMP-9

**Figure 5:**
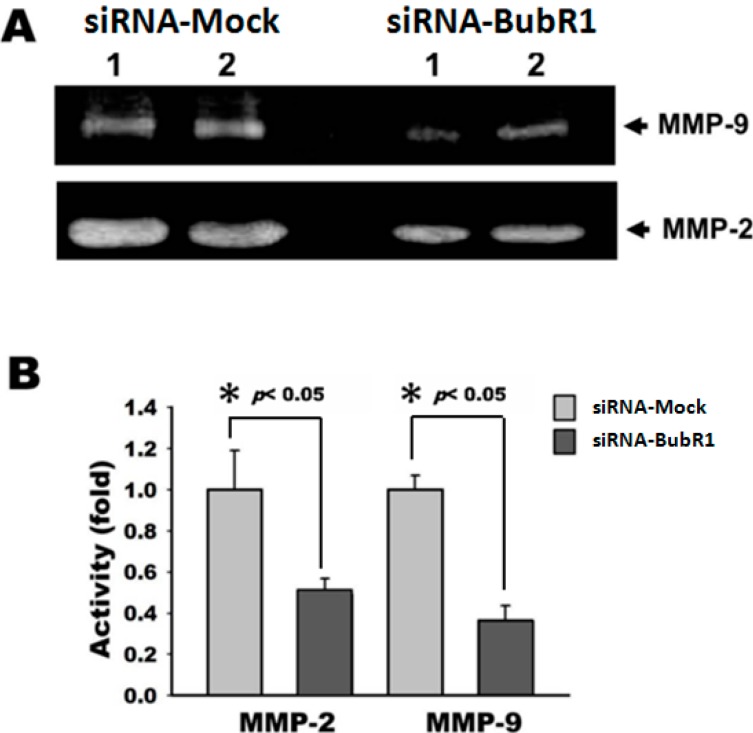
Knockdown of BubR1 attenuates the activity of MMP-2 and -9. (**A**) Ca9-22 cells with BubR1-siRNA and Mock-siRNA were incubated with serum-free medium for 24 h, respectively, and the supernatant mediums were harvested to determine the activity of secreted MMP-2 and MMP-9 by gelatin zymography; and (**B**) The quantitative results of unstained gelatin-degradation zones. Each representative blot was performed in at least triplication. * *p* < 0.05 for Mock *vs.* siRNA-BubR1.

MMP-2 and -9, both of these enzymes are subjected into gelatinases, principally due to their ability to structural remodel the space of ECM [21]. Numerous studies have demonstrated that down-regulation or inactivation of MMP family, especially MMP-2 and MMP-9, weakens the local invasive ability of cancer cells presenting at the boundary of primary tumors [22]. Because knockdown of BubR1 decreased the cell migration of Ca9-22 cells, we tested whether knock-down of BubR1 could also down-regulate the activity of MMP-2 and MMP-9. Our results showed that, in zymography assays, knockdown of BubR1 disabled the secretory proteins of Ca9-22 cells to digest the gelatins as the mock did (Figure 5), indicating that MMP-2 and MMP-9 might be modulated by BubR1-mediated OSCC progression.

### 2.6. The Overexpression of BubR1 May Involve in the OSCC Tumorigenesis

**Figure 6:**
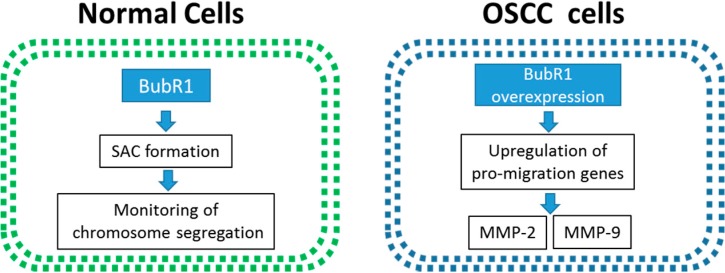
Proposed role of BubR1 in the tumorigenesis of OSCC. In normal cells, BubR1 interacts with other proteins to form SAC for regulating chromosome segregation during mitosis. However, in oral cancer cells, the overexpression of BubR1 alternatively causes the up-regulated expression of certain migration-associated genes, such as MMP-2 and MMP-9 metalloproteinases. Eventually, this promotes the migration of oral cancer cells, and may facilitate the migration ability, a hallmark of cancer metastasis of OSCC.

Previous studies have shown that BubR1 acts as a monitor of genome integrity for preventing aneuploidy. However, the mechanism of BubR1-promoted cancer progression remains controversial. Our results showed that BubR1 was highly overexpressed in all five tested OSCC cell lines compared to oral fibroblast cells, HOK and HGF (Figure 1 and Figure 2). Up-regulation of mRNA levels of BubR1 in OSCC cell lines prompted us to establish whether this had any oncogenic function connected to cancer progression, especially metastasis. The result of wound healing assays revealed that Ca9-22 cells migrated to the wound site after 12 h and BubR1 knockdown slowed down about 30% of the migratory property of Ca9-22 cells, and knockdown of BubR1 significantly inhibits about 60% of invasion ability. In addition, MMP-2 and -9, which had a high correlation with cancer metastasis, was significantly suppressed by BubR1 siRNA. Based on the previous study, the relevance of mRNA levels of BubR1 and the migration ability of OSCC cell lines are positively correlated. These above findings suggest that the pathological role of BubR1 in promoting the metastasis of OSCC cells (Figure 6).

Other investigations have noted that the adaptor protein Ajuba, which had been described in regulating the cell adhesion and migration processes, served as a microtubule-associated protein (MAP) that interacted with BubR1 to maintain the accuracy of chromosome segregation during mitosis [23]. The function of MAPs not only involves managing the spindle microtubules in mitotic checkpoint, but also in the organization of microtubules network in vital process, such as construction of track for transporting the cellular cargos and controlling the flexibility of cytoplasm for cellular movement [24].

## 3. Experimental Section

### 3.1. Cell Cultures

Primary culture of normal human oral keratinocytes (HOKs) from the oral mucosa was obtained during dental surgery. HOK cells were cultured in defined keratinocytes-SFM (Life Technologies, Carlsbad, CA, USA) supplemented with epidermal growth factor (EGF) and bovine pituitary extract. Human normal gingival fibroblast (HGF) and four human OSCC cell lines including Ca9-22 (gingival carcinoma), HSC-3 (tongue carcinoma), FaDu (hypopharyngeal carcinoma), SCC-9 (tongue carcinoma), SAS (tongue carcinoma) and Cal-27 (adenosquamous carcinoma) were cultured in Dulbecco’s modified Eagle’s medium (DMEM) (Gibco, Grand Island, NY, USA) and supplemented with 10% fetal bovine serum (FBS), 100 units/mL penicillin, 100 μg/mL streptomycin, 0.03% glutamine, and 1 mM sodium pyruvate. Cells were incubated at 37 °C in a humidified atmosphere containing 5% CO_2_.

### 3.2. Quantitative Polymerase Chain Reaction Analysis

Total RNA were prepared by TRIzol reagent (Life Technologies), following the manufacturer’s instruction. In the first strand reaction, denaturation of the mRNA was performed by mixing 4 μg RNA, 1 μL oligo (dT), and 1 μL dNTP mixture (dATP, dGTP, dCTP, dTTP, all of them were at 10 mM) in 10 μL ddH_2_O at 65 °C for 5 min. Subsequently, the complimentary DNA (cDNA) was synthesized by in reverse transcriptase buffer with Moloney murine leukemia virus reverse transcriptase (Promega, Madison, WI, USA) at 37 °C for 50 min. RNase H was added to remove RNA away from the new-created cDNA and further stored at −80 °C. Quantitative polymerase chain reaction (qPCR) was performed using the Step One System (Applied Biosystems, Foster City, CA, USA) according to manufacturer’s instruction. Primer sequences for qPCR are listed at Table 1. One microliter cDNA was mixed with 10 μL Power SYBR Green PCR Master Mix (Applied Biosystems), 1 μL forward and reverse primer (10 mM) in total 20 μL ddH_2_O. The cycling condition for all target genes is 95 °C for 10 min, followed by 40 cycles of 95 °C for 15 s and 60 °C for 1 min. Glyceraldehydes-3-phosphate dehydrogenase (GAPDH) gene was also examined as an internal RNA control. The expression levels were normalized to GAPDH reference to obtain the relative threshold cycle (*C*_t_).

**Table 1:** Primers designed for real-time PCR in the study.

Genes	Primers
*BubR1*	Fʹ: 5ʹ-GCCTCGTGGCAATACAGCTT-3ʹ
	Rʹ: 5ʹ-TGGTGTCATAACTGGCTGTTGTG-3ʹ
*gapdh*	Fʹ: 5ʹ-GTCTTCACCACCATGGAGAA-3ʹ
	Rʹ: 5ʹ-ATGGCATGGACTGTGGTCAT-3ʹ

### 3.3. Immunofluorescence Staining

The 1 × 10^4^ cells were seeded and on the glass slide, which had been steeped in 67% Nitric acid for the cell adhesion. Use of 4% paraformaldehyde (in PBS) fixed the cell shape for 5 min when the confluent of the cells achieved to 70%. The fixed cells were permeabilized by 0.5% Triton x-100 (in PBS), then using 1% bovine serum albumin (BSA) for blocking. After 1 h, the cells were incubated with primary antibody at 4 °C overnight. Thereafter, cells were washed by 1% BSA 3 times, at 5 min intervals, and further incubated with Alexa Fluro 555/FTC-conjugated secondary antibodies for 1 h. At the last 10 min, Alexa Fluor^®^ 594 phalloidin (Cat# A12381, Molecular Probes, Eugene, OR, USA) using for staining filaments-actin and the nuclei staining dye 4′,6-diamidino-2-phenylindole (DAPI) were added. The over-stained cells were washed by 1% BSA 3 times, at 5 min intervals. Finally, the stained cells were dropped by mounting medium for amplifying the fluorescence signal and further observed by a confocal fluorescence microscope.

### 3.4. RNA Interference

The BubR1 siRNA oligonucleotide duplexes were purchased from Dharmacon (Lafayette, CO, USA). The siRNA duplexes targeted to the mRNA of human BubR1 (Sequence ID: NM_001211.5). The sequences of the BubR1 siRNA were as follow: Sense strand as 5′-AACAAUACUCUUCAGCAGCAG-3′ and anti-sense strand as 5′-CUGCUGCUGAAGAGUAUUGUU-3′. Two OSCC cells, Ca9-22 and Cal-27, were transfected with siRNAs using 8 μL INTERFERin™ siRNA transfection reagent (Cat# 409-10, Polyplus Transfection, New York, NY, USA) according to the manufacturer’s protocol in a 6-well format after seeding for 24 h. The transiently transfected cells were assayed after transfection for 48 h.

### 3.5. Cell Proliferation Assay

Cell proliferation assays were performed after both Ca9-22 and Cal-27 cells transiently transfected with the BubR1-siRNA or the Mock-siRNA, respectively. Briefly, the cells were plated in a 12-well plate at 1 × 10^5^ cells per well and incubated at 37 °C in a humidified atmosphere containing 5% CO_2_ for 48 h. After incubation, cells were exposed to 0.2% trypan blue were counted by Countess™ automated cell counter (Invitrogen, Carlsbad, CA, USA).

### 3.6. Western Blotting

Western blot assay was conducted as described previously [25]. Briefly, the cells transiently transfected with the BubR1-siRNA or the Mock-siRNA were harvested and lysed. Lysates were centrifuged, and protein concentrations were determined using a Bicinchoninic acid (BCA) Protein Assay Kit (Pierce, Rockford, IL, USA). The 40 μg protein lysates were separated by 10% SDS-polyacrylamide gel electrophoresis and then electrotransferred. The transferred membranes (PALL, Ann Arbor, MI, USA) were incubated with a primary antibody against BubR1 (Cat# 612502, BD Transduction Laboratories™, San Diego, CA, USA), MMP-2 (Cat# 29575, AnaSpec, San Jose, CA, USA), MMP-9 (Cat# 53678, AnaSpec), and GAPDH (Cat# GTX627408, GeneTex, Irvine, CA, USA), and the corresponding secondary antibodies. The ECL™ (Amersham Piscataway, NJ, USA) chemiluminescence detection kit was used for signal detection.

### 3.7. Wound Healing Assay

Transfected cells were seeded and grown in a 12-well plate until forming a full monolayer. The cells were then scratched by 200 μL tip of pipet to create a straight wound and washed twice with PBS. Thereafter, the scratched cells were incubated with 8% serum contained media at 37 °C for 12 h to reconstruct the wound site. The wound gaps were photographed and analyzed by freeware “TScratch” [20].

### 3.8. Transwell Invasion Assay

The assessment of the cellular motility was described previously with minor modifications [17]. Forty-eight hours after transiently transfecting Ca9-22 and Cal-27 cells with the BubR1-siRNA or the Mock-siRNA for 48 h, respectively. One hundred thousand transfected cells were cultured in 200 μL serum-free medium, which subsequently are added into the upper compartment of the Boyden’s chamber with 8-μm filter pores (ThinCerts™-TC Inserts, 24 well). The 8% fetal bovine serum is used in the lower chamber as the chemoattractant. After 12 h for cell invasion, the non-invaded cells on the upper surface of the filter are removed by cotton bud with methanol, and the cells on the lower surface of the filter are fixed with 0.5% formaldehyde and stained with Giemsa stain (Merck, Darmstadt, Germany). Cells from the whole region of each insert were counted under an inverted microscope (TE2000-U; Nikon, Tokyo, Japan) equipped with NIS-Elements Software (Nikon).

### 3.9. Gelatin Zymography

The activities of MMP-2 and MMP-9 were tested by gelatin zymography as previously described [26]. Briefly, 3 × 10^5^ of Ca9-22 cells were seeded onto 12-well plates and transfected with BubR1-siRNA or Mock-siRNA for 48 h. Afterward, these transfected cells were further incubated with serum-free DMEM/F12 (1:1 ratio) for 24 h. The supernatants were collected and subjected to electrophoresis with 10% SDS polyacrylamide gels containing 1% gelatin. Gels were washed twice with distilled water containing 2.5% Triton x-100 to remove SDS and subsequently incubated at 37 °C for overnight for reactivation of gelatinase activity. The gels were stained with Coomassie brilliant blue R-250 and then destained with methanol-acetic acid-water (50/75/875, *v*/*v*/*v*). The gelatinase activity was acquired by measuring the unstained gelatin-degradation zones within the intensities of gel signal were analyzed using Gel Pro v.4.0 software (Media Cybernetics, Silver Spring, MD, USA).

### 3.10. Statistical Analysis

All data were presented as means ± S.D. The Student’s *t*-test analysis was used to test the mean difference between two groups.

## 4. Conclusions

These studies suggest that BubR1 may cooperate with other proteins such as MAPs to promote the cell migration of OSCC. Besides, BubR1 expression could serve as a marker to distinguish the invasiveness from patients with a OSCC tumor after the primary tumor has been surgically removed. The present results of our study shed the light on the promoter role of BubR1 in the progression of OSCC, especially the cellular invasion and migration.
